# Meshless optical mode solving using scalable deep deconvolutional neural network

**DOI:** 10.1038/s41598-022-25613-4

**Published:** 2023-01-19

**Authors:** G. Alagappan, C. E. Png

**Affiliations:** Institute of High-Performance Computing, Agency for Science, Technology, and Research (A-STAR), Fusionopolis, 1 Fusionopolis Way, #16-16 Connexis, Singapore, 138632 Singapore

**Keywords:** Optics and photonics, Computational science

## Abstract

Optical mode solving is of paramount importance in photonic design and discovery. In this paper we propose a deep deconvolutional neural network architecture for a meshless, and resolution scalable optical mode calculations. The solution is arbitrary in wavelengths and applicable for a wide range of photonic materials and dimensions. The deconvolutional model consists of two stages: the first stage projects the photonic geometrical parameters to a vector in a higher dimensional space, and the second stage deconvolves the vector into a mode image with the help of scaling blocks. Scaling block can be added or subtracted as per desired resolution in the final mode image, and it can be effectively trained using a transfer learning approach. Being a deep learning model, it is light, portable, and capable of rapidly disseminating edge computing ready solutions. Without the loss of generality, we illustrate the method for an optical channel waveguide, and readily generalizable for wide range photonic components including photonic crystals, optical cavities and metasurfaces.

## Introduction

Machine learning^1,2^ techniques are widely used in image classification^3^, natural language processing^4^, automatic speech recognition^5^ and robotics^6^. Apart from these traditional applications, machine learning methods have recently penetrated vast areas of science and engineering fields which are conventionally explored by deterministic hard computing methods. In photonics, machine learning techniques have been used in improving the state of the art in optical fiber sensing, laser characterization, quantum communications, optical imaging, photolithography, inverse designs of photonic devices with targeted performances, and on-demand designs of metamaterials^7–14^. In this article we tackle the optical mode solving problem for the general parameter space of photonics using deep learning methods.

Optical mode solving is of paramount importance in photonic design and discovery. Traditionally, this is handled by solving time independent Maxwell equations^15–17^. It is usually the first step in any photonic device design, and engineers usually spent plenty of their design time in this step extracting and optimizing mode distributions, field confinements, coupling coefficients, effective and group refractive indices. Optical modes of a particular photonic geometry also serve as basis functions in the analysis of perturbative effects such as sensing, defect in nano-cavities, waveguide tapers and waveguide gratings. Therefore, accurate and rapid mode-solving are extremely necessary to have an accelerated photonic model. Apart from the speed, the mode solution also must be memory wise light and portable so that they can be easily deployed as edge or mobile computing solutions^18–20^. This will greatly benefit many photonic systems such as optical measurement devices, lidars, photonic integrated circuits, and fiber-based communications. Edge computing aims to deliver computing paradigm closer to the source of data. To appreciate this, consider the fabrication process of a photonic waveguide, as an example. Once the waveguide is fabricated, the optical metrological devices will measure the geometrical parameters. The edge computing if available at the measurement device, it will enable instantaneous prediction of the mode profiles at the point of geometrical measurements.

Analytical solutions for optical modes exist for simple one-dimensional slab waveguides. For two-dimensional geometries such as channel, and strip waveguides, photonic crystals, and metasurfaces numerical simulations must be sought for accurate solutions. Usually, to tackle the numerical problem, one applies mesh-based methods such as finite difference, and finite element^15–17^. These methods discretize the entire computational geometry into finite number of meshes. The number of meshes increases with the size of the geometry. Alternatively, mesh free methods such as spectral^21,22^ and finite cloud^23^ methods are available. In spectral method, the computational geometry is divided into a set of structurally invariant domains. The fields inside the domain are expanded using a pre-selected set of basis functions. In the finite cloud method, instead of domains, clouds of nodes are distributed throughout the geometry. The density of the nodes can be controlled as per anticipated field gradients. In all these methods (with or without mesh), one finally encounters matrix diagonalization to solve for unknown coefficients and fields. The size of the matrix directly scales with the number of meshes (in mesh-based method) and number of domains/nodes in the abovementioned mesh-free methods. Although, matrix diagonalization methods are well established, they consume a significant amount of computational resources, and this hinders design acceleration when performing a large number of geometrical sweeps, and optimizations.

The photonic parameter space is finite and well defined. We know the wavelengths, the exact materials, and the range for the fabrication feasible geometrical dimensions of the devices. Researchers often explore the well-defined parameter space repeatedly with brute force numerical methods. In the current perspective of machine learning era, such repeated exploration is thus, an inefficient use of computational resources. Any finite parameter space can be captured, and an effective representation can be formulated. Such representation often assumes deep learning models and have been proven to be a solid replacement for the traditional photonic models^24–33^.

In this article, we show that an effective deep learning representation for the optical mode solving can be built for the usual parameter space of photonics^34^. The field profile of an optical channel waveguide is solved using a deep deconvolutional neural network (DCNN). We illustrate a meshless method, with full resolution scalability that is generic in photonic wavelengths, materials, and geometrical dimensions. The proposed method is readily generalizable for finding mode images in photonic crystals, optical cavities, nanostructures and metasurfaces. It shares ubiquitous benefits of deep learning model of being light, portable, fast and edge computing ready solutions. Furthermore, it can provide an excellent set of basis functions for ultraquick evaluations of advance numerical algorithms that tackles perturbative problems. One such example includes the eigenmode expansion method^35^, which is often used to design waveguide tapers and waveguide Bragg gratings. Here, the waveguide modes are used as basis function to evaluate the effect of perturbations (i.e., grating or taper profiles). The basis modes traditionally are evaluated using the time-consuming numerical methods. This is clearly a bottleneck in the entire eigenmode expansion algorithm and can be easily fixed by replacing the traditional solver with the deep learning-based mode solvers.

**Figure 1:**
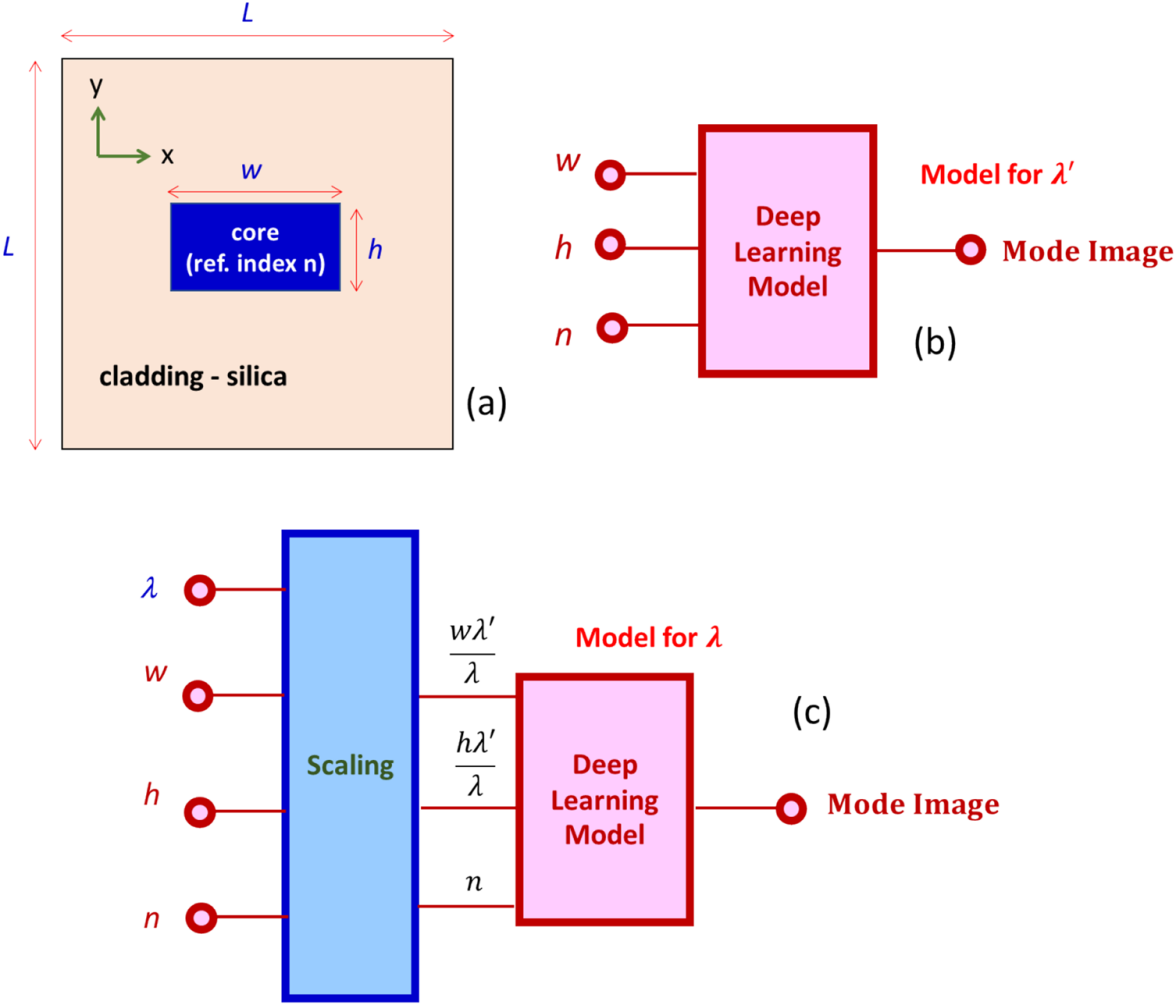
(**a**) Schematic of the optical channel waveguide. (**b**) The deep learning model trained at λ′ = 1.55 μm. (**c**) The wavelength generic deep learning model.

Figure 1a shows the schematic of the optical channel waveguide. The waveguide has a rectangular geometry with width, *w* and height, *h*. We consider a fabrication feasible photonic parameter space for *w* and *h*. The spans of *w* and *h* are 0.15 μm < *w* < 1.1 μm, and 0.1 μm < *h* < 0.5 μm. Silica is used as a cladding material as it the popular choice for a wide range of applications. The core refractive index is taken as *n*, and the span 1.6 < *n* < 4 covers refractive indices of most photonic materials. The prediction domain has a square shape with sides of *L* = 1.5 µm. The model is designed to predict the magnetic field pattern of the fundamental waveguide mode the with transverse electric (TE) polarization. Fundamental TE modes are used for most of the on-chip applications. A total of 3000 data points is prepared using commercially available Lumerical MODE software^36^. To save the computational time in Lumerical MODE software, we applied anti-symmetric and symmetric boundary conditions along *x* and *y* directions (see axes definition in Fig. 1a), respectively. For details on the symmetric and anti-symmetric boundary conditions, please see Ref.^37^.

In Lumerical MODE simulation, the size of the computation domain is not fixed to *L* = 1.5 µm. The computational domain for numerical calculation is kept sufficiently large to ensure proper decay of the evanescent field. The computational window for low refractive index contrast waveguides (i.e., longer evanescent tail) are much larger than computational window for high refractive index contrast waveguides (i.e., shorter evanescent tail). However, during the data preparation stage the mode images are truncated with *L* = 1.5 µm. This will help to standardize the size of the prediction window, and the output mode images. The value of *L* = 1.5 µm is conveniently picked such that it is larger than the maximums of *w* and *h* in the input parameter space. All data points are checked for their convergence and existence of a sensible mode. The associated effective refractive indices of all mode profiles in the data set are also verified independently with our earlier high precision deep learning models that predicts the effective refractive indices^32^. Therefore, the DCNN is trained with only convergent solutions of Maxwell equations within the specified parameter space. As a result, it will only output convergent solutions for inputs within the boundaries of the input parameter space.

Firstly, we built our deep learning model at the telecommunication wavelength (*λ*′) of 1.55 μm. The block diagram is shown in Fig. 1b. The input is a three-dimensional vector comprising *w*, *h* and *n*, and the output is an image (a matrix) representing the distribution of the field value in the square domain of size *L*, with a resolution of *N* by *N* pixels. The model built for wavelength *λ*′ can be used for a generic wavelength* λ* by having a scaling layer as shown in Fig. 1c. Here we apply the scaling property of Maxwell’s equation. There is no fundamental length scale in Maxwell’s equations which fortuitously allow devices to be modelled at different wavelengths by simply scaling the geometrical dimensions in proportion to the change in wavelengths. For an example, if you would like to find mode image at *w* = *w*_*o*_, *h* = *h*_*o*_ for a wavelength *λ*_*o*_ using the model developed for* λ*′, then the geometrical inputs to the *λ*′ model must be scaled by quantity* λ*′/*λ*_*o*_. The model presented in Fig. 1c is able to handle the refractive index dispersion of the core material. Varying refractive index is now can be set for different wavelengths [i.e., *n*(*λ*)].

**Figure 2:**
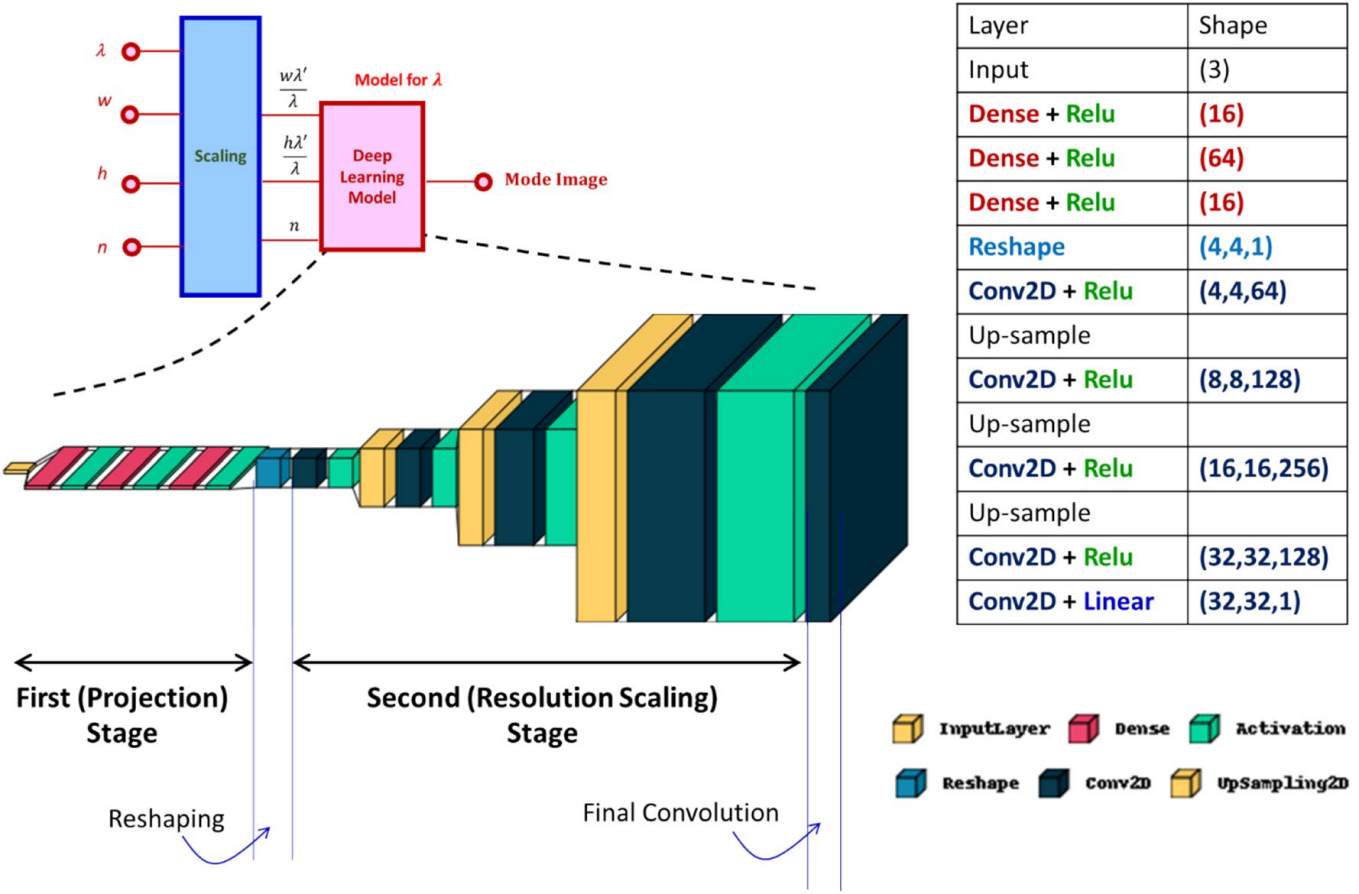
The DCNN architecture. It has two stages, projection, and resolution scaling. The key on the right bottom, labels the layers of different colors. The table in the figure elaborates the details of the layers and their shapes.

In Fig. 2, we reveal the architecture of the deep learning model embedded in the block of Fig. 1b. As the input is a vector and the output is an image, the architecture must be able transform the vector to a matrix. We accomplish this by mean of a DCNN. The proposed DCNN has two stages performing projection and resolution scaling. The first stage projects the three-component vector into a higher dimensional space, resulting in a vector of *D*^2^ components. This stage comprises fully connected feedforward layers with rectified linear unit (ReLU) activations^38,39^. In training deep neural networks, vanishing gradient is a common issue. ReLU activation helps circumventing this problem. The expression for ReLU is *f*(*x*) = max(0, *x*). It is a piecewise linear function that outputs the input directly if it is positive, otherwise, outputs zero. ReLU is the most common activation in CNN and DCNN since Alexnet discovered in 2012^40^.

We used *D* = 4. This means the 3-component vector is transformed into a 16 (*D*^2^) component vector at the end of stage one. Before entering the second stage, the vector with *D*^2^ components is reshaped to a *D* × *D* matrix. The second stage scales the resolution to the required *N* × *N* pixels, and it has multiple blocks of up-sampling and convolution layers with varying filter (channel) size. The kernel size of the sliding convolution window is 3 by 3. At the end of the second stage, we have a final convolution layer with linear activation. It collapses all the filters in the earlier layer and produces the final image. The trainable parameters consist of neurons (first stage) and filters (second stage). As higher resolution is handled using an additional resolution-scalable block (convolution block) in the second stage, the higher resolution does not constitute to more neurons. However, the extra convolution block increases the total numbers of filters that to be trained. The extra learning is addressed effectively via a transfer learning approach and will be elaborated later in this article.

**Figure 3:**
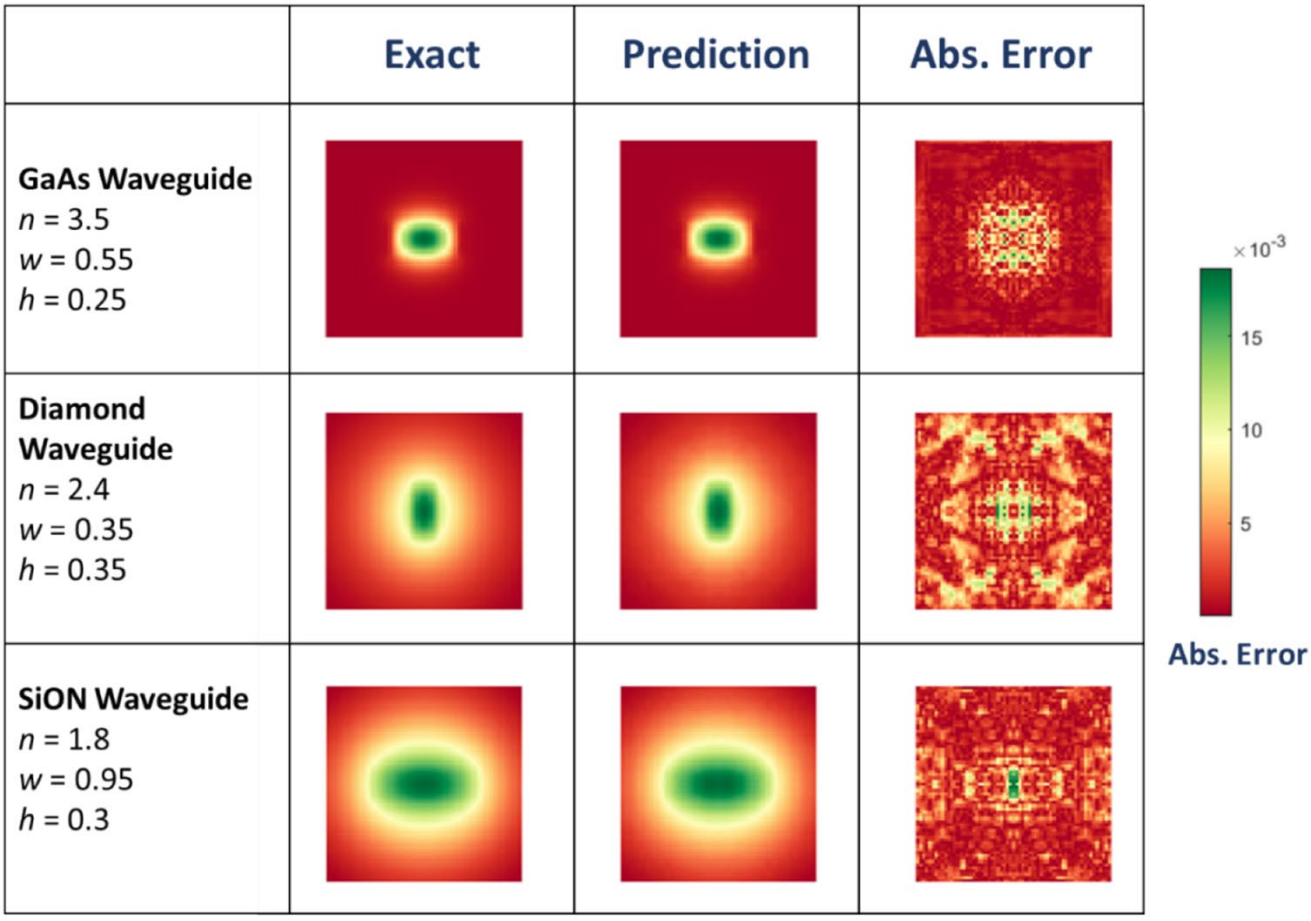
The glimpses of DCNN ability in predicting the mode images of various photonic waveguides. The second column and the third column showcase the exact and predicted (with DCNN architecture in Fig. 2) mode patterns respectively. The third column plots the absolute error at every pixel.

**Figure 4:**
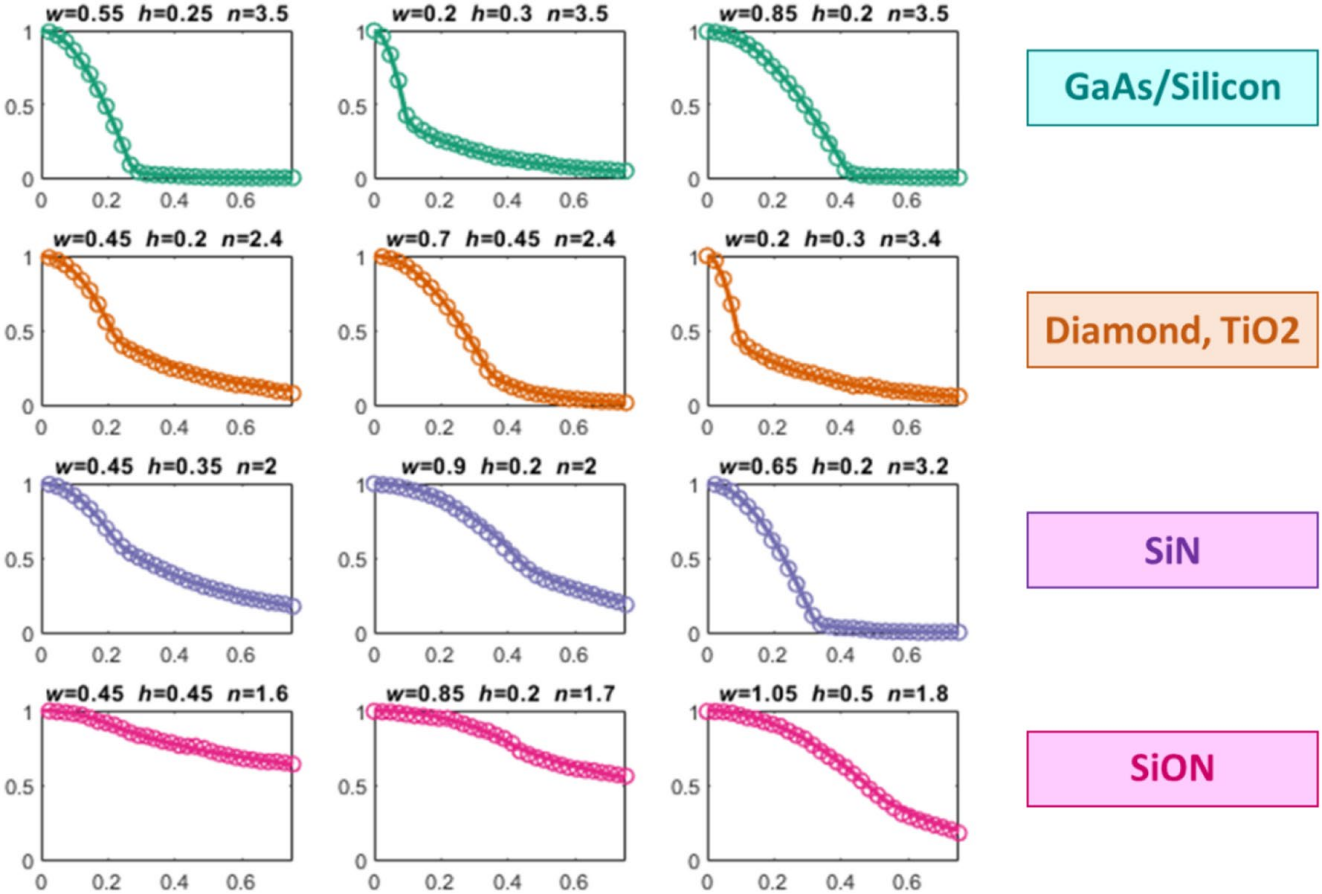
The horizontal cutlines of various photonic channel waveguides. Solid lines—exact numerical computation. Circles—prediction with DCNN architecture in Fig. 2.

In Figs. 3 and 4, we present the glimpses of DCNN ability of producing high quality mode images and cutlines for waveguides constructed with various core materials. The first row in Fig. 3 demonstrates exact and predicted mode images of gallium arsenide (GaAs) waveguide with an aspect ratio of more than 1 [*w*/*h* > 1]. Here the mode is tightly confined to the core of the waveguide. The second row in Fig. 3 showcases a square diamond waveguide, and the third row displays similar mode patterns for a silicon-oxynitride waveguide with a weak mode confinement. As can be visually seen, the predicted and exact mode patterns are in very good agreement. The third column in Fig. 3 displays the absolute error, |*H*_exact_ − *H*_predict_| with *H*_exact_ and *H*_*predict*_ being the exact and predicted values of the magnetic field at each pixel.

Note the depicted mode profiles in Fig. 3 will also apply for a lot of other photonic materials with similar refractive indices such as silicon, titanium oxide and polymers. The exact mode image is obtained via numerical solution to Maxwell’s equation using the method finite differences. We used the commercially available Lumerical MODE solver. The deep learning predicted image comes from the DCNN model illustrated in Fig. 2. The fine detail of the architecture is elaborated in the insert of the Fig. 2. Both *H*_exact_ and *H*_*predict*_ are normalized such that the maximum value is 1. Figure 4 shows the field values for *y* = 0 (i.e., along the horizontal cutlines in Fig. 3) for various waveguides. The example comprises various cases such as different aspect ratio, weak and tight confinements. Again, we can readily verify from Fig. 4, both predicted and exact mode profiles are in very good agreement for various waveguide geometries and material systems.

**Figure 5:**
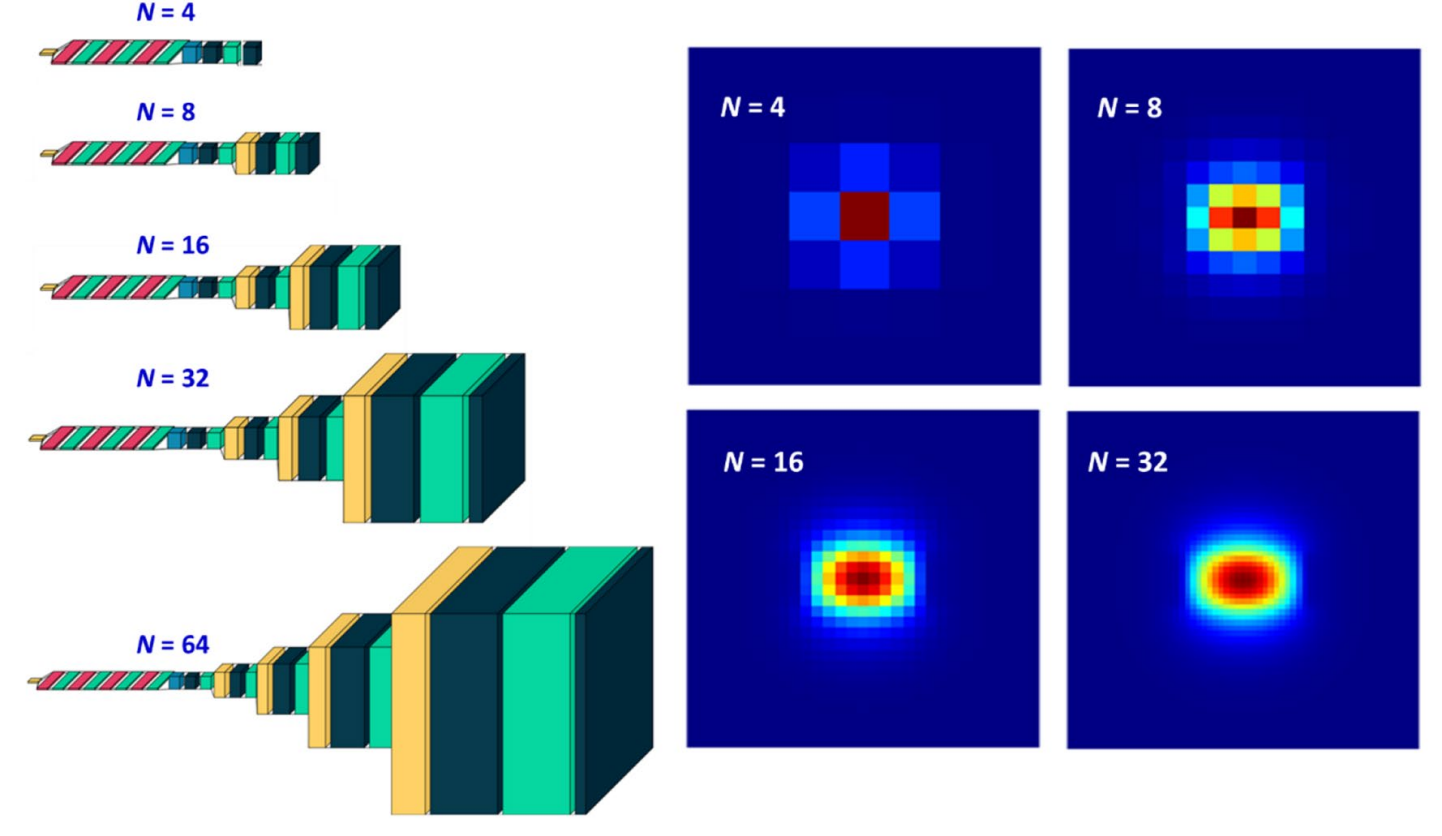
Scalable resolutions via DCNN. (**a**) DCNN for various resolutions. (**b**) The electric field pattern of the fundamental waveguide mode in a silicon waveguide with *w* = 0.55 μm, *h* = 0.25 μm, *n* = 3.5.

Figure 5 portrays the resolution scaling mechanism in DCNN. This is done with a series of scaling blocks. Each scaling block consist of up-sampling, two-dimensional convolution and ReLU activation layers. For a final image with *N* × *N* pixels, a total of 1 + log_2_(*N*/*D*) scaling blocks is required. For the first scaling block immediately after the first stage, the up-sampling layer is substituted by a reshaping layer. The up-sampling layer increases the dimensionality of the matrix, while the convolution–activation combo learns the fine features of the mode distribution. In the left panel of Fig. 5 we showcase the field patterns of the fundamental mode silicon waveguide (*w* = 0.55 μm, *h* = 0.25 μm, *n* = 3.5), generated by DCNN architectures of varying *N*. The number of filters (*n*_*filters*_) in each scaling block is an hyperparameter, and the optimization is carried out by means of a transfer learning approach. We start with training architectures of smaller *N*. The trained weights are transferred [i.e., copied and kept frozen] when training the architecture with a larger *N*. In the architecture with larger *N*, only the newly inserted scaling block is trained, and in this manner both precision and training time can be optimized.

**Figure 6:**
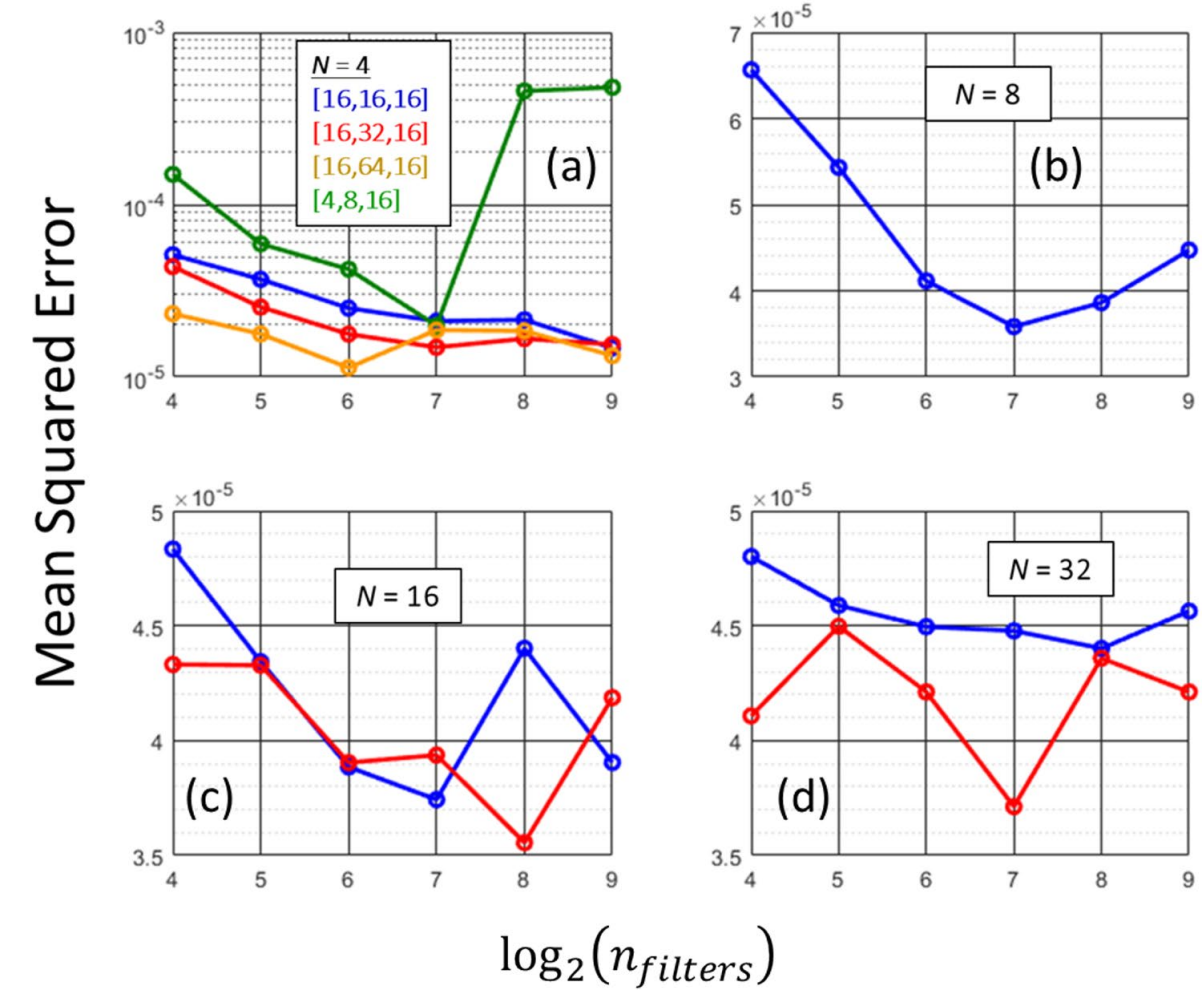
Optimization of number of filters (*n*_*filters*_) in DCNN architectures of varying resolutions, *N*. (**a**) Case *N* = 4. Mean square error as a function *n*_*filters*_ for various configurations of feedforward connection. (**b**) Case *N* = 8. Mean square error as a function* n*_*filters*_. (**c**) Case *N* = 16. Mean square error as a function *n*_*filters*_. (**d**) Case *N* = 32. Mean square error as a function *n*_*filters*_. In (**b**–**d**), the blue curves represent mean square errors for the optimization of final scaling block with frozen inner block weights. The red curves represent learning with fine tuning, where all the scaling blocks participates in the training.

The training of DCNN is done using a root mean squared backpropagation algorithm with a learning rate of 0.001^38^. The data set is split into training, validation and testing sets in a ratio of 0.7, 0.2 and 0.1, respectively^39^. In deep learning framework bulk portion of the data will be used for model building (i.e., training—determination of weights). The major fraction of the remaining data will be used for validation. The validation data points are not used in training, but they are used to monitor the validation loss which signals overfitting of the deep learning model. Finally, a small set of data of will be reserved for testing. The testing data set is kept totally aside from training and validation, and it is used to independently measure and compare the performance of various hyperparameters. As a rectangular symmetry prevails in all mode images, in the training, we used only the quarter of the actual image where there is no symmetry present. We begin training with an architecture of *D* = *N* = 4. For the feedforward connections, we used three hidden layers and considered four sets of neuron distributions. As *D* = 4, the number of neurons in the last hidden layer is kept at *D*^2^ = 16. The first set has a neuron distribution of 16 × 16 × 16 [i.e., 16 neurons in each layer]. The second and third sets have distributions of 16 × 32 × 16 and 16 × 64 × 16. The last set has a progressive distribution of 4 × 8 × 16. Figure 6a shows the mean square error in the test data set as a function number of filters in the convolutional layers. From Fig. 6a, we can see that the optimized architecture has a feedforward connection with 16 × 64 × 16 neurons and *n*_*filters*_ = 64. The progressive distribution [4 × 8 × 16] performs poorer than the other distributions.

For training architecture with an higher resolution *N* = 8, we transfer the learning from the optimized architecture of *N* = 4. The architecture for *N* = 8 replicates the optimized architecture of *D* = *N* = 4 with copied pre-trained weights and, an extra scaling block at the end for resolution increment [see Fig. 5]. The training and optimization of *N* = 8 architecture is done only for this extra scaling block. Figure 6b shows the mean square error as a function *n*_*filters*_ of the last scaling block. As can be readily seen, the optimal value for number of filters in the extra scaling block is 128. In the similar manner the optimizations for *N* = 16, and 32 architectures are carried out by mean of the described transfer learning and the results are shown in blue curves of Fig. 6c,d. The red curves represent the fine-tuning case when the training of inners scaling blocks are allowed. As we can see Fig. 6d, the final fine-tuned model reaches a mean squared error of 3.7 × 10^–5^. This, error translates to precision 2 to 3 decimal places in absolute value. Such error is small^27^, and visually leaves no discrepancy between predicted and exact mode patterns (see Fig. 3).

In order to assess the resource efficiency of the presented model, we quantified the computational efforts taken by the DCNN model and compared it with the traditional finite difference method. The DCNN model is written in python, and thus for a fair assessment we made a comparison with a finite difference optical mode solving code written in the same programming language^41^. We performed DCNN and finite difference simulations for all geometries in the testing data set. Testing data set consists of 332 geometries (i.e., combinations of *w*, *h* and *n*) randomly sampled in the input parameter space. The resolution in finite difference simulation is kept the same as in the DCNN model [*N* = 32, *n*_*filters*_ = 128]. The computational domain in finite difference is taken as *Sw* and *Sh* in *x* and *y* directions, respectively. Here, *S* is a is scaling factor and *S* should be sufficiently large for proper convergence. In DCNN model, as we elucidated earlier, during the data preparation stage the computational domain was kept sufficiently large, and every data point is ensured its convergence with a sensible mode. Using ASUS laptop with intel i7 CPU [1.8 GHz, 4 cores 8 GB RAM], the DCNN model took ~ 14 s to predict the mode profiles of all the 332 test geometries. In the finite difference model, the model consumed ~ 76, ~ 140, and 230 s for the cases of *S* = 3, 4 and 5, respectively. DCNN calculations are therefore 5-, 10-, and 16-fold faster than the finite difference simulations with *S* = 3, 4 and 5, respectively. These results portray clear advantages of DCNN in terms of the computational speed.

Throughout this article we used rectangular channel waveguide with isotropic core material as an example. However, the presented meshless, resolution scalable mode solving method is universal, and can be adopted for many photonic devices by modifying the input vector. In the case of directional couplers (i.e., two closely spaced parallel waveguides), apart from the waveguide parameters (*w*, *h* and *n*) the spacing between the two waveguides must be supplied in the input vector. For the case of anisotropic materials, the orientation of the principal axis must be supplied, and single refractive index used for isotropic materials must be substituted with principle refractive indices. For photonic crystals, the input vector must consist of the parameters that describes the geometry of the unit cell and the period of the lattice. In all these devices, architectures of projection stage and resolution scaling stage will remain the same. The number of neurons, feedforward layers, and convolutional filters can be tuned with the help of the presented transfer learning approach.

Simple shapes like circles, squares, and rectangles with perfect geometries are often the best starting point in most photonic device designs. However fabricated structures will have some irregularities. These irregularities can be captured, parameterized, and included in the input vector for optical mode solving. For devices with totally irregular shapes, a different approach is required. One can use autoencoder^39^ kind of architecture, in which the neural network is supplied with the input image of the waveguide and train it to produce to mode profiles. Though promising, the implementation of such structure requires a huge data set and effort in training.

In summary we have presented a novel deep learning architecture that is capable of predicting mode images of an optical channel waveguide with high precisions. The input to the model is a vector containing the geometrical and material parameters of the waveguide. The internal architecture of the model make uses a deep deconvolutional neural network, and functions in two stages. The first stage transforms the input to a vector in higher dimensional space, and the second stage reshapes the vector to a matrix and performs resolution scaling. The presented deep learning architecture is generic for a prediction of a specific mode with a pre-defined polarization and symmetry. In this article, we illustrate training and predictions of fundamental TE waveguide modes. To predict fundamental transverse magnetic (TM) waveguide modes, one has to simply retrain the model with TM data sets, and optimize the hyperparameters (i.e., number of neurons and filters). For multimode waveguides, the presented architecture has to be adopted for each different modes and the boundaries of parameter space have to be re-defined to include the mode cut-offs.

The mode effective refractive index can be obtained from feedforward deep learning models^32^ with high accuracies. These architectures are extensively described in our earlier papers. As mode profiles, and effective refractive indices are related to each other, in future, predictions of these two quantities can be combined. This can be potentially done using a dual-output deep learning architecture and employing Keras functional APIs^38,39^.

## Data Availability

The datasets used and/or analysed during the current study available from the corresponding author on reasonable request.
